# Depressive mood ratings are reduced by MDMA in female polydrug ecstasy users homozygous for the l-allele of the serotonin transporter

**DOI:** 10.1038/s41598-018-19618-1

**Published:** 2018-01-18

**Authors:** K. P. C. Kuypers, R. de la Torre, M. Farre, L. Xicota, E. B. de Sousa Fernandes Perna, E. L. Theunissen, J. G. Ramaekers

**Affiliations:** 1 Department of Neuropsychology and Psychopharmacology, Faculty of Psychology and Neuroscience, Maastricht University, Maastricht, The Netherlands; 2 Integrative Pharmacology & Neurosciences Systems Research Group, Institut Hospital del Mar d’Investigacions Mèdiques, Barcelona, Spain; 3 Universitat Autonoma de Barcelona, Barcelona, Spain; 4 Hospital Universitari Germans Trias i Pujol, Clinical Pharmacology, Badalona, Spain; 5 Spanish Biomedical Research Centre in Physiopathology of Obesity and Nutrition (CIBEROBN), Santiago de Compostela, Spain; 6 Universitat Pompeu Fabra, CEXS-UPF, Barcelona, Spain

## Abstract

MDMA exerts its main effects via the serotonergic system and the serotonin transporter. The gene coding for this transporter determines the expression rate of the transporter. Previously it was shown that healthy individuals with the short allelic variant (‘*s*-group’) of the 5-HTTLPR-polymorphism displayed more anxiety and negative mood, and had a lower transcriptional efficiency compared to individuals who are homozygous for the *l*-allele (‘*l*-group’). The present study aimed to investigate the role of the 5-HTTLPR polymorphism in MDMA-induced mood effects. Four placebo-controlled, within-subject studies were pooled, including in total 63 polydrug ecstasy users (N_s-group_ = 48; N_l-group_ = 15) receiving MDMA 75 mg and placebo on two test days, separated by minimally 7 days. Mood was assessed by means of the Profile of Mood States. Findings showed that MDMA induced –independent of sex- a positive mood state, and as a side effect also increased two negative affect states, anxiety and confusion. Anxiety ratings were higher in the *l*-group and independent of treatment or sex. Depression ratings were lowered by MDMA in the female *l*-group. Findings indicate that the MDMA-induced reduction in self-rated depressive feelings is sex- and genotype-dependent, with females homozygous for the *l*-allele showing this beneficial effect.

## Introduction

MDMA is known as a ‘chemically messy’ drug, implying that it works on a whole range of neurotransmitters. It exerts its main effects on the monoamines with most pronounced effects on the central nervous serotonin (5-HT) system^1^. While there is some evidence for direct agonistic action at specific 5-HT receptors, it is known that the serotonin transporter (SERT, 5-HTT) is a major target for MDMA^1–3^. Acutely, MDMA causes the reuptake transporter to work in reverse and thereby causes an increase in synaptic 5-HT^2^. MDMA has been shown to induce effects on mood, increasing in general positive (e.g., vigor, arousal, elation) as well as negative affective states (e.g., confusion, anxiety)^4,5^. Studies have demonstrated a role for the serotonin transporter in these effects since MDMA effects on mood and consciousness were counteracted by blocking of the 5-HTT^6^.

The serotonin transporter gene (*SLC6A4*) encodes the serotonin transporter. A polymorphism in the promoter region of this gene (5-HTTLPR) influences its transcriptional activity and regulates 5-HTT expression and density in the brain. The 5-HTTLPR is a repeat polymorphism with long (*l*) and short (*s*) alleles. The long subgroup has a higher density of 5-HTT (30–40%) and approximately a double of 5-HT uptake capacity compared to s-allele carriers^7,8^. The s-allele is associated with lower 5-HTT expression and function and higher levels of anxiety and negative mood in healthy individuals as well as smaller amygdala volumes and increased amygdala reactivity which has been shown to be inversely related to the amygdala volume^9–12^.

During the last decade, the interest in using MDMA as an adjunct to psychotherapy in the treatment of psychiatric conditions such as post-traumatic stress disorder has grown. Preliminary data have also shown the efficacy of this treatment^13,14^. Of importance for these patient studies is that MDMA induces, next to elevated positive affect, also an increase in negative affect such as anxiety and confusion, in healthy volunteers^4^. Since it previously has been shown that carriers of the s-allele show heightened fear and anxiety^15^ it is of interest to investigate how s-allele carriers respond emotionally to a dose of MDMA compared to l-homozygotes. This knowledge could feed into potential tailored MDMA-assisted therapy, based on genetics.

The aim of the present study was to investigate whether the MDMA-induced mood effects differ between ecstasy users who are s-carriers and those who are homozygous for the l-allele. It is hypothesized that ecstasy users carrying the slow working variants of the SERT genotype will acutely release less 5-HT via the SERT and consequently experience less pronounced mood effects immediately after use compared to homozygous l-allele carriers who are known to have a greater efficacy in transporting serotonin^16^. Since typical sample sizes of placebo-controlled MDMA studies, large enough to detect behavioral effects, are too small to detect effects at the genotype level, data from 4 placebo-controlled studies were pooled^5,17–19^.

## Results

### Mood

#### Positive affect

**Figure 1:**
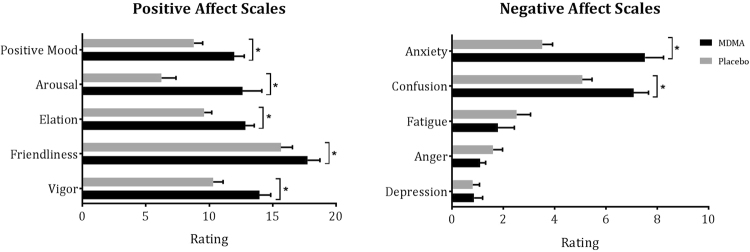
Mean (±SE) ratings on positive and negative affect scales of the POMS. *Depicts statistically significant Treatment effects (p ≤ 0.05).

GLM analysis revealed a main effect of Treatment on all the positive mood scales. Under influence of MDMA participants felt more vigorous (*F*_1,59_ = 18.21, *p* < 0.001, *ƞ*_*p*_^2^ = 0.24), more friendly (*F*_1,59_ = 6.01, *p* = 0.02, *ƞ*_*p*_^2^ = 0.09) more elated (*F*_1,59_ = 20.71, *p* < 0.001, *ƞ*_*p*_^2^ = 0.26) more aroused (*F*_1,59_ = 16.33, *p* < 0.001, *ƞ*_*p*_^2^ = 0.22) and they experienced more positive mood (*F*_1,59_ = 15.77, *p* < 0.001, *ƞ*_*p*_^2^ = 0.21) (Fig. 1). There were no effects of Sex, Genotype, or their interaction with Treatment on the positive mood scales.

#### Negative affect

**Figure 2:**
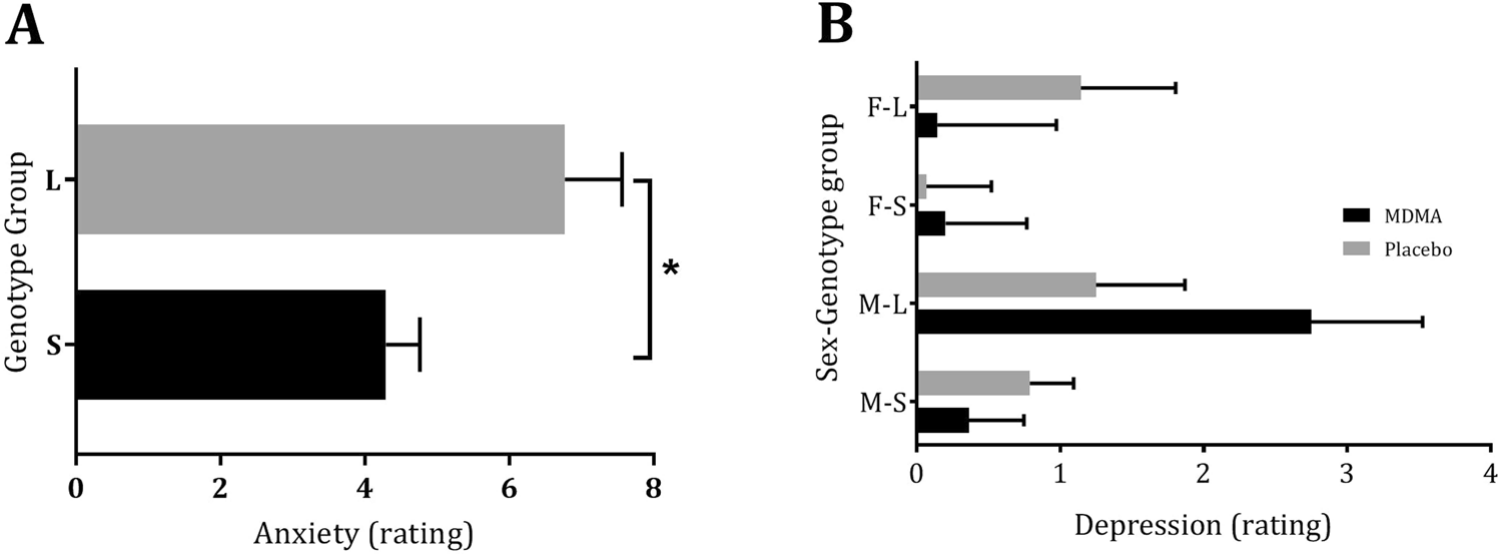
Mean (±SE) anxiety ratings for genotype by sex groups (**A**) and depression ratings by sex and genotype group (**B**). *Indicates a statistically significant effect of genotype at p ≤ 0.05; F = Female; M = Male; S = *s/l and ss*-carriers group, L = *l/l*-carriers group.

GLM analysis revealed a main effect of Treatment on 2 negative affect scales, i.e., under influence of MDMA participants reported higher levels of anxiety (*F*_1,59_ = 31,82, *p* < 0.001, *ƞ*_*p*_^2^ = 0.35) and confusion (*F*_1,59_ = 12.41, *p* = 0.001, *ƞ*_*p*_^2^ = 0.17) (Fig. 1). A main effect of Genotype (*F*_1,59_ = 7.29, *p* = 0.009, *ƞ*_*p*_^2^ = 0.11) was demonstrated on one negative affect scale, i.e. Anxiety, showing that anxiety ratings were higher in the L-group compared with the S-group. There was a Treatment by Sex by Genotype interaction effect on Depression (*F*_1,59_ = 5.56, *p* = 0.02, *ƞ*_*p*_^2^ = 0.09). The response pattern in males in both genotype groups was the complete opposite of that of females in both genotype groups. Univariate GLM analysis showed that the difference between depression ratings under MDMA and placebo of both female genotype groups was statistically significant (*F*_1,39_ = 4.76, *p* = 0.04, *ƞ*_*p*_^2^ = 0.19); for males this difference approached significance (*F*_1,39_ = 3.78, *p* = 0.059, *ƞ*_*p*_^2^ = 0.09). Depression ratings of females in the placebo condition differed significantly (*F*_1,20_ = 5.07, *p* = 0.04, *ƞ*_*p*_^2^ = 0.20) between the s- and l-group. Findings seem to indicate that MDMA reduces the higher depression ratings in the female L-group compared to the S-group while the opposite (though not statistically significant) was observed in males (Fig. 2). There were no effects of Treatment, Sex, Genotype or their interactions on Anger and Fatigue.

### MDMA concentration

**Figure 3:**
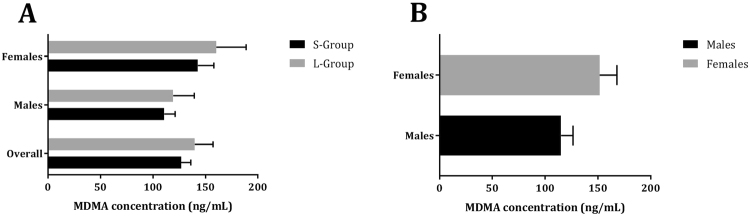
Mean (±SE) MDMA concentrations (ng/mL) per genotype (**A**) and sex (**B**).

Univariate ANOVA did not reveal main effects of sex (*F*_1,1_ = 66.87, *p* = 0.08, *ƞ*_*p*_^2^ = 0.98), genotype (*F*_1,1_ = 8.49, *p* = 0.21, *ƞ*_*p*_^2^ = 0.89), or their interaction (*F*_1,52_ = 0.05, *p* = 0.82, *ƞ*_*p*_^2^ = 0.01) (Fig. 3).

### Correlation between mood and MDMA concentrations

Pearson’s correlation analysis showed that only one POMS scale significantly correlated with MDMA concentrations, i.e., anxiety ratings were positively related with MDMA concentrations (*r*_56_ = 0.26, *p* = 0.05). The other scales were not statistically significant correlated with MDMA concentrations in blood (Table 1).

**Table 1:** Pearson’s correlations between POMS scales and MDMA concentrations in blood (MDMA BC). *Indicates statistically significant interaction at *p* ≤ 0.05.

Positive affect and MDMA BC	Vigor	Friendliness	Elation	Arousal	Positive Mood
*r*_56_; *p*	0.17; 0.22	0.13; 0.36	0.16; 0.24	0.20; 0.14	0.14; 0.30
**Negative affect and MDMA BC**	**Depression**	**Anger**	**Fatigue**	**Confusion**	**Anxiety**
*r*_56_; *p*	−0.01; 0.94	0.08; 0.57	−0.14; 0.30	0.20; 0.14	0.26; 0.05*

## Discussion

The aim of the present study was to investigate the role of the serotonin transporter gene in MDMA-induced mood effects. In addition, it was tested whether effects differed between sexes and whether MDMA mood effects correlated with MDMA blood concentrations. Findings showed that MDMA, in line with previous findings, induced a positive mood state and elevated feelings of anxiety and confusion. One main effect of genotype was revealed, i.e., individuals in the *l*-group felt, irrespective of sex or treatment, more anxious compared to the *s*-group. In general, genotype did not seem to influence the MDMA effects, except for one affective state, i.e., depression. It was demonstrated that self-rated subjectively experienced ‘depression’ was attenuated by a single dose of MDMA in the female l-group.

The latter findings on *l*-genotype groups and negative affect seem counterintuitive since most studies have shown that *s*-carriers have higher levels of anxiety and are more at risk for developing depression compared to homozygous *l*-individuals^11,12^. The question rises whether this group of ecstasy users is representative of the ‘general’ population with respect to the genotype. Looking at the 5-HTTLPR distribution which is 76.2–23.8% for the *s*- and *l*-group respectively in the present sample it can be concluded that the sample of ecstasy users did not differ from the general European population with a distribution of 68% for the s-group and 32% for the *l*-group^9^. Previously, it was shown by another study that there was a slight tendency for a higher frequency of homozygous *s*-individuals in an ecstasy user group, although the differences were not statistically significant and samples were in the Hardy–Weinberg equilibrium^20^. On the other hand, it has previously been shown that ecstasy users who were *s*-allele carriers showed abnormal processing on an affective Go-NoGo paradigm and had elevated depression scores on a questionnaire, when compared with a control group^21^, suggesting that ecstasy users display aberrant emotion-related behavior compared to a drug naïve and cannabis user group. However, a depression baseline pattern comparable to our findings has previously been shown in non-drug using participants^22^ and higher anxiety levels were reported by *l/l*-carriers in the presence of a stressor^23^. With respect to the latter it might be postulated that participants in double-blind, placebo-controlled, experimental MDMA studies experience the situation as ‘exciting’ since they know there is a possibility that they will receive MDMA in an unusual (laboratory) setting. However, this is speculative and needs further investigation. With respect to the question whether this group of ecstasy users is representative of the (cognitive) ‘healthy’ population, we have demonstrated previously that the same or comparable population of ecstasy users was not memory impaired compared to a healthy control group^24^.

Interestingly, the literature on the 5-HTTLPR is not that consistent as it seems and since a few years it has been acknowledged that the level of methylation of the transporter may also be a source of diverging outcomes^25,26^. Associations between 5-HTTLPR polymorphisms and psychological problems are significantly altered by environmentally induced methylation patterns of the transporter^27^. Early and recent stress has been shown to lead to hyper-methylation^28^ and higher levels of methylation of the 5-HTT were for example associated with increased risk of unresolved responses to loss or trauma in the otherwise ‘protective’ homozygous *l*-variant of the 5HTTLPR. MDMA can be seen as a biological stressor, causing a substantial increase in cortisol concentrations^17^ and therefore potentially affecting methylation. Yubero *et al*.^29^ demonstrated that MDMA indeed influences the methylation of the 5-HTT independent of genotype. MDMA (75 mg) caused, 165 minutes after intake, an increase in serotonin transporter expression, which was independent of genotype^29^. Interestingly, findings suggested that changes could be more pronounced in women and in *l/l*- carriers of the 5-HTTLPR genotype since the expression in females increased about 100% compared to only 50% in males and homozygous individuals are known to have increased 5-HTT expression. However, since there was no genotype by sex interaction effect in the methylation study, confirmation for this suggestion is pending. Another study showed that women had statistically significant more pronounced physiological effects after MDMA administration compared to males and high functionality 5-HTTLPR individuals demonstrated increased cardiovascular effects^30^. Partly in line with these findings, i.e., suggesting that females and *l/l-*carriers are more sensitive to MDMA effects, the present study demonstrated that the effects of MDMA on depression ratings were only visible in female l-group individuals. Of note, the effect size of the interaction effect was small and the sample size of the female l-group was <10, therefore current results should be handled with care and not be over-interpreted.

Previously, Oakly and colleagues (2014) suggested that individuals with a lower SERT activity would be more sensitive to the reinforcing properties of MDMA^31^. The absence of a disproportionate subjective reaction to MDMA-induced positive mood effects in *s*-carriers of the 5-HTTLPR in the present study seems to counter this suggestion. Previously, it was also shown that MDMA effects on emotional empathy were positively related with MDMA blood concentrations^32^. In the present study, anxiety levels correlated positively to MDMA blood concentrations which is consistent with a previous study showing anxiety to be present when the dose of MDMA was higher^33^. The other mood states were unrelated to MDMA blood concentrations indicating that a single fixed dose induces the same mood effect in users. However, since only one dose of MDMA was used in the present study, variation in MDMA concentrations is suggestible lower than when multiple doses are included. Current findings therefore do not allow the exclusion of the possibility that MDMA concentrations and mood states, other than anxiety, are associated.

In the present study, a bi-allelic determination of the 5-HTTLPR was done while a tri-allelic determination is also possible and might provide more information. One variant of the long allele of the 5-HTTLPR, ‘*l*_A_’ is associated with high levels of 5-HTT mRNA transcription and ‘*l*_G_’ is more similar to ‘*s/s*’ with low transcriptional and expressive levels 5-HTT^34^. In the tri-allelic distribution in the white population, 25% are *l*_A_/*l*_A_ individuals and thus classified as in the ‘long’ group, whereas 6% is *l*_G_/*l*_G_ and thus categorized in the ‘short’ group^35^. For the present study this implies that less than 1 individual could be misclassified, assuming that the distribution of the 5-HTTLPR is the same in the ‘ecstasy users population’ compared to the non-drug users population. The effects of this are therefore suggested to be minimal, though it would be advisable that future MDMA studies include the tri-allelic determination in larger samples consisting of both sexes.

Findings indicate that the MDMA-induced reduction in self-rated depressive feelings is sex- and genotype-dependent, with females homozygous for the *l*-allele of the 5-HTTLPR showing this beneficial effect although this effect was small. MDMA effects on other positive and negative mood states seem to be independent of sex, 5-HTTLPR genotype and MDMA bloods concentrations. This suggests that there is no need to take genotype or sex into account when treating patients with MDMA since these groups demonstrated the same emotional response to MDMA. Replication in larger samples sizes is recommended.

## Methods

### Participants

Four placebo-controlled within-subject studies investigating the effect of MDMA on mood were included in the present pooled data analysis. The studies were all individually approved by the Medical Ethics Committee of the Academic Hospital of Maastricht and Maastricht University^5,17–19^ and conducted in accordance with the Declaration of Helsinki. All the trials were registered in the Dutch Clinical Trial Registry (http://www.trialregister.nl/trialreg/index.asp): study 1: NTR 1421 (26/08/2008), study 2: NTR2636 (03/12/2010), study 3: NTR2792 (24/02/2011), study 4: NTR3691 (08/11/2012). Participants provided written informed consent to participate in the studies and were paid.

Inclusion criteria were age between 18–40 years, free from medication, an absence of psychiatric history (personal or first-degree relative) and any major medical, endocrine or neurological conditions, good medical health as determined by a medical history and examination, blood analyses, and ECG, minimally three experiences with ecstasy/MDMA, normal weight, body mass index (weight/height^2^) between 18 and 28 kg/m^2^, and written informed consent. Exclusion criteria were history of drug abuse (other than the use of MDMA) or addiction, for women: pregnancy or lactation, no use contraception, excessive drinking (>20 alcoholic consumptions a week), hypertension (diastolic > 100; systolic > 170)^5,17,18^. When participants in the separate studies met the criteria and were physically and mentally healthy as determined by questionnaires and a medical examination, the experimenter assigned a participant number which was linked to a treatment sequence. The treatments were randomized using a Latin Square. Both the participant and the experimenter were blind to the treatment order.

The pooled sample size included 72 polydrug ecstasy users. They were all mentally and physically sound as determined by questionnaires and a medical examination. There were missing genotyping data for participants due to difficulties with blood drawing; this resulted in a final sample of N = 63. Lifetime prevalence of the included participants of the following drugs was: 87% for cannabis, 19% for amphetamine, 32% for cocaine, 46% for magic mushrooms, 3% for LSD and 16% for ‘other’ drugs (ketamine, GHB, Ritalin and salvia). Additional demographic information of the final sample is given per study in Table 2.

**Table 2:** Characteristics of participants (Mean [±SD]) and methods per study.

Study number	Demographics					Reference
	Sample size (M/F)	Age (years)	Ecstasy/MDMA use (times used in life)	5-HTTLPR		
				S-group (M/F)	L-group (M/F)	
1	17 (11/6)	21.0 (1.2)	17.0 (33.0)	13 (8/5)	4 (3/1)	17
2	15 (9/6)	21.1 (2.2)	11.1 (9.9)	11 (8/3)	4 (1/3)	18
3	14 (11/3)	22.1 (1.9)	17.0 (12.7)	12 (10/2)	2 (1/1)	5
4	17 (10/7)	21.1 (2.7)	18.0 (25.0)	12 (7/5)	5 (3/2)	19

### Procedure

After study inclusion and before actual test days, participants were familiarized with the study procedure and questionnaire on a training day. They were requested to abstain from any drug use 1 week before the medical examination until the last test day. Participants were asked not to use any caffeinated or alcoholic beverages 24 hours before testing and to get a normal night’s sleep as assessed.

Prior to experimental sessions at 9 AM participants were screened for drugs of abuse (THC, opiates, cocaine, amphetamines, methamphetamines), and had to pass a breathalyzer ethanol test. Women were given a pregnancy test. When tests were negative, participants had breakfast, and a blood sample was taken to determine the 5-HTTLPR genotype afterwards. This was followed by administration of the study treatment. The mood questionnaire and a second blood sample were taken 90 minutes after treatment. Test days were minimally separated by a 7-day wash-out period.

Study treatment consisted of identically looking placebo or MDMA (75 mg) capsules, administered orally under double-blind conditions. The 75 mg dose was chosen because previous research showed that it can elicit effects on mood and behavior^36,37^. A permit for obtaining, storing, and administering MDMA was obtained from the Dutch drug enforcement administration.

### The Profile of Mood States

The Profile of Mood States (POMS)^38^ is a self-assessment mood questionnaire with 72 five point-Likert scale items, representing eight mood states; i.e., Anxiety, Depression, Anger, Vigor, Fatigue, Confusion, Friendliness and Elation. Two extra scales are derived, i.e. Arousal ((Anxiety + Vigor) − (Fatigue + Confusion)) and Positive mood (Elation − Depression). The participant has to indicate to what extent these items were representing his/her mood.

### Genotype

A blood sample was collected in order to determine the bi-allelic genotype of the 5-HTTLPR. Sample genotyping was done by extracting DNA from peripheral blood leukocytes of participants using the Flexi Gene DNA kit (Qiagen Iberia, S.L., Spain) and applying a previously described protocol^39^. Subjects were split according to genotype and associated 5-HTTLPR functionality. Due to the distribution of the genotypes among participants, alleles were grouped in two main clusters, i.e., the *l*-group (homozygous for the *l*-allele) and the *s*-group (homozygous or heterozygous for the *s*-allele: *s/l* and *s/s*).

### MDMA concentration

A blood sample was centrifuged immediately and the resulting plasma was stored at −20 °C until analysis. MDMA was determined by gas-chromatography coupled to mass spectrometry using a method previously described by Pizarro and colleagues^40^.

### Statistical analysis

POMS data entered a general linear model (GLM) repeated measures procedure (SPSS, version 24.0) with Treatment (2 levels, MDMA/Placebo) as within subject factor, and Genotype (2 levels, *s*-group/*l*-group) and Sex (2 levels) as between subject factors. In case of interaction effects, difference scores between MDMA and placebo conditions were calculated and Univariate GLM analysis was conducted. MDMA concentration data entered a univariate analysis of variance (ANOVA) with Sex (2 levels) and Genotype (2 levels) as between subject factors. Pearson’s correlations were calculated between POMS scales and MDMA concentrations.

The alpha criterion level of statistical significance for all analyses was set at *p* = 0.05, uncorrected, this in order to avoid Type II errors with small sample sizes;. Effect sizes are reported as partial eta-squared (*ƞ*_*p*_^2^) to demonstrate the effect’s magnitude where 0.01 is defined as small, =0.09 as medium, and 0.25 as large. Partial eta squared is based on Cohen’s *f* which defines small, medium and large as respectively 0.10, 0.25, and 0.50^41^.

### Data availability

The datasets generated during and/or analysed during the current study are available in the Dutch Dataverse Network: https://dataverse.nl/dvn/dv/NP.
